# Aspergillosis infection over 20 years: a case report of probable vascular invasion in central nervous system

**DOI:** 10.1186/s12883-020-01919-6

**Published:** 2020-09-11

**Authors:** Yong Zhang, Xu Wu, Yang-Pan Hu

**Affiliations:** Department of Neurology, Renmin Hospital of Wuhan University, Wuhan, 430060 China

**Keywords:** Headache, Central nervous system infection, *Aspergillus*, Voriconazole, Vascular invasion, Case report

## Abstract

**Background:**

Aspergillosis infection of central nervous system (CNS) is rare and fatal. Diagnosis of invasive aspergillosis remains difficult. Aspergillosis of CNS can be an acute, subacute, or chronic onset, and the longest course of the disease was currently reported to be 4 years. Here, we report a case with recurrent headache over 20 years.

**Case presentation:**

A 54-year-old man was admitted to our neurological disease ward due to intermittent throbbing headache lasting for more than 20 years that had grown notably worse over the past week. The headache was localized to the side of his head blown by a cold wind. He also experienced nausea and vomiting when the headache became severe. The headache usually lasted for 3–4 h after he was blown by the cold wind, though he had no fever. The neurological examination was normal. Magnetic resonance imaging (MRI) of the brain was negative for parenchymal and meningeal lesions. However, the case had increased intracranial pressure (ICP), and elevated protein level in the cerebrospinal fluid (CSF). *Aspergillus fumigatus* was found in CSF by nanopore targeted sequencing (NTS) and in blood by enzyme-linked immunosorbent assay (ELISA). *Aspergillus fumigatus*-specific antibody IgG was 104.62 AU/mL, aspergillus galactomannan (GM) antigen was 3.27 μg/L, D-dimer was 3.25 mg/L and fibrinogen degradation product was 11.50 mg/L, which were markedly higher than the normal levels. The patient was prescribed by voriconazole. After the treatment of 14 days, the ICP, CSF protein level, *Aspergillus fumigatus*-specific antibody IgG, GM antigen, D-dimer and fibrinogen degradation product returned normal. *Aspergillus* was disappeared by NTS test of CSF. His headache has never occurred again after blowing by a cold wind.

**Conclusions:**

This report reveals that aspergillosis infection of CNS can last for more than 20 years and the major symptom is only intermittent throbbing headache in an immunocompetent patient. Vascular invasion is probably the mechanism of headache in our case with CNS aspergillosis infection. Performing high-throughput gene sequencing technology in CSF is important when the pathogen is not determined for the patients with suspected CNS infection.

## Background

Aspergillosis infection of central nervous system (CNS) is rare and it happened in the patients with a disease of highly immunocompromised persons including those with prolonged neutropenia, recipients of hematopoietic stem-cell transplants or solid-organ transplants, and patients with advanced acquired immunodeficiency syndrome or chronic granulomatous disease [1]. Usually, the involvement of the CNS is a devastating consequence of disseminated aspergillosis and may be manifested by headache, seizures or focal neurologic signs from mass effect or stroke. Diagnosis of invasive aspergillosis remains difficult in that clinical manifestations are not specific and cultures of sample lack sensitivity [1, 2]. The wide application of high-throughput gene technology highly increases the possibility that unexpected pathogens are revealed. At the same time, the technology enlarges the acknowledgment of the clinical symptoms of aspergillosis infection. Here, we report a case of aspergillosis infection of CNS over 20 years.

## Case presentation

On October 30, 2019, a 54-year-old man was admitted to our neurological disease ward due to intermittent throbbing headache lasting for 20 years that had grown notably worse over the past week. Twenty years earlier, he started to have intermittent throbbing headache after severe influenza. The patient complained his headache was always triggered by cold wind and it was localized to the side of his head blown by a cold wind. He also experienced nausea and vomiting when the headache became severe. The headaches usually lasted for 3–4 h after he was blown by the cold wind, though he had no fever. The weather had been cold for the past week, so the frequency and severity of his headaches had increased. So, he was afraid to use air-conditioning in hot summer and he must wear thick hat and scarf in winter when he went outside. He claimed to have no other significant medical history than chronic hepatitis B. Because his headache was tolerable and not severe, he occasionally used painkillers.

He was awake and alert with normal mental status. His vital signs were within normal limits. Cranial nerve examination was unremarkable. Movement and sensation were symmetrical in his extremities. His neck was supple with a negative Kernig’s sign and Brudzinksi’s sign. Lumbar puncture was performed, but intracranial pressure (ICP) was not measured. The number of white blood cells (WBCs) in the cerebrospinal fluid (CSF) was 1/μL. There was no atypical cell. The lymphocyte ratio and monocyte ratio were 0.69 and 0.31, respectively. The sugar level was 50 mg/dL (serum 90 mg/dL) and the protein level was moderately high at 510 mg/L. Ink staining, acid-fast staining, and culture were negative. A positive result was shown for *Aspergillus fumigatus* by nanopore targeted sequencing (NTS) of CSF (kit provided by Wuhan Dgensee Clinical Laboratory Co. Ltd., Wuhan, China; Laboratory at Wuhan University People’s Hospital, Wuhan, China). The reads for *Aspergillus fumigatus* was 1176 by NTS. D-dimer was 3.25 mg/L and fibrinogen degradation product was 11.50 mg/L. These were markedly higher than the normal levels. The conventional and contrast enhanced magnetic resonance imaging (MRI) of the brain was negative for parenchymal and meningeal lesions. Additional examination showed a negative blood routine, sedimentation rate, liver and kidney function, cellular immunity, and humoral immunity. The patient was advised to inject fluconazole at the local hospital, 0.2 g each time, twice a day, for 14 days. He did so, but his headaches persisted. The patient returned to our neurological clinic on November 19. Lumbar puncture was performed in the outpatient department, but ICP was still not measured. The protein level was increased to 1090 mg/L. Blood tests for *Aspergillus* (Wuhan Kangsheng Global Co., Ltd., Wuhan, China) revealed IgG antibody to *Aspergillus fumigatus* 104.62 AU/mL, *Aspergillus* galactomannan (GM) antigen 3.27 μg/L, which were markedly higher than the normal levels. The patient was re-admitted to the hospital on December 9. On the day of the admission, lumbar puncture indicated an ICP of 240 mmH_2_O, WBCs 2/μL, lymphocyte ratio 0.63, monocyte ratio 0.35, glucose concentration 50 mg/dL, and protein concentration 590 mg/L. Ink staining, acid-fast staining, and CSF culture were negative. The cryptococcal antigen was negative. So, he was diagnosed as probable aspergillosis infection of central nervous system. He received voriconazole 0.2 g each time, twice a day from December 10. On December 15, repeat tests showed *Aspergillus* IgG 45.92 AU/mL and GM antigen < 0.25 μg/L, which were normal. After 7 days of treatment, he had nausea and his liver function was mildly impaired. Entecavir and magnesium Isoglycyrrhizinate was prescribed to protect his liver function. On December 23, his headache relieved significantly and nausea was disappeared. Lumbar puncture was repeated and showed a normal CSF analysis: ICP 170 mmH_2_O, white blood cell count 2/μL, lymphocyte ratio 0.45, monocyte ratio 0.55, glucose level 65 mg/dL, and protein level 430 mg/L. *Aspergillus* was negative by NTS test of CSF. The liver function was normal. He was discharged on the same day. It was recommended that he continue oral voriconazole tablets, 0.2 g each time, twice daily for 10 weeks. On January 16, 2020, he returned to the clinic without a headache for 20 days. The D-dimer and fibrinogen degradation product were 0.35 mg /L and 4.3 mg /L, respectively, which were both normal. On March 26, 2020, we followed up with him, he has stopped taking voriconazole tablets for 3 weeks and had no headache for 3 months. He was satisfied with the treatment.

## Discussion and conclusions

*Aspergillus* is an opportunistic pathogen. *Aspergillus* infection is more common in immunocompromised people such as transplant recipients, long-term immunosuppressant users, and people with diabetes, persistent neutropenia, and advanced human immunodeficiency virus infection. The patient had no other significant medical history than chronic hepatitis B and he was immunocompetent. Aspergillosis of the central nervous system (CNS) can be an acute, subacute, or chronic onset [2], and the longest course of the disease was currently reported to be 4 years [2]. The patient had a chronic headache of more than 20 years, which is the longest reported course so far. Most patients with *Aspergillus* infection in CNS manifested as meningitis and brain parenchyma damage. Unlike infection by *Candida*, infection by *Aspergillus* rarely involved fever or signs of meningeal irritation, WBCs and level of protein and glucose in the CSF were usually lower or even within normal limits [3]. The patient only showed mild abnormalities such as headache, increased ICP, and elevated protein level in the CSF. The mild clinical symptoms of the patient were consistent with slightly abnormal results of CSF analysis. The mild clinical symptoms lasted for more than 20 years without progression, which may be related to the patient’s immunocompetence.

*Aspergillus fumigatus* is the most common pathogen of invasive aspergillosis [2]*,* and it was found in the CSF and in the blood of our case. The pathological mechanism of invasive aspergillosis is the vascular invasion with subsequent tissue infarction and necrosis [4]. The patient had intermittent throbbing headache induced by cold wind, which might be due to the vasoconstriction and blood flow stasis caused by the cold. In addition, the D-dimer and fibrinogen degradation product were higher than normal before the treatment. After voriconazole treatment, the clinical symptoms disappeared, and D-dimer and fibrinogen degradation product also returned to normal levels. The changes of D-dimer level and fibrinogen degradation product level further supported the pathological mechanism of cerebrovascular involvement in CNS aspergillosis. At the same time, the signs of meningeal irritation and the conventional and contrast enhanced MRI of the brain were negative, which was helpful to exclude the involvement of parenchymal and meningeal lesions. Kousha M et al. thought the patients with pulmonary aspergillosis may also present with pleuritic chest pain due to vascular invasion [5]. Therefore, involvement of cerebral vascular is probably the mechanism of headache in the patient with aspergillosis infection. In terms of the route of infection in our case, we thought hematogenous dissemination was possible. On the one hand, CNS aspergillosis may occur by hematogenous dissemination in the setting of disseminated infection, or by direct extension from the ear, paranasal sinuses, or mastoids in patients with localized invasive aspergillosis [6]. On the other hand, severe influenza infection can develop concurrently with invasive aspergillosis in apparently immunocompetent individuals [7]. Because the patient developed headache after severe influenza, and the *aspergillus fumigatus* was observed in CSF and blood, and the vascular invasion was probably the key pathological mechanism of our case. Besides, our case had no localized head aspergillosis. Therefore, we thought hematogenous dissemination was the probable route in our case.

The diagnosis of CNS aspergillosis is mostly based on the positive CSF culture or biopsy or culture of brain tissue to identify the pathogen. However, the rate of positive culture has been reported to be very low [2, 8]. The patient was a middle-aged male with atypical clinical symptoms and laboratory test results. The course of the disease was over 20 years. His CSF culture is negative. Based on the clinical presentations of the patient, abnormal level of protein and abnormal ICP in CSF, the diagnosis of CNS infection was highly suspected, so the Aspergillus culture test and NTS was carried out in CSF. After observing positive results for *Aspergillus fumigatus* by the NTS in CSF, we initially questioned the results. Because of our inexperience, fluconazole was prescribed to patients. As a result, it was ineffective. At last, the patient returned to our hospital again. We further performed the blood GM antigen test to confirm *Aspergillus* infection. Of course, limited by our medical service, we can’t perform the GM antigen test in CSF. After voriconazole treatment, the patient’s headache disappeared. The ICP and CSF protein level returned normal, which further supported the clinical diagnosis of the CNS aspergillosis. NTS is a rapid and convenient high-throughput gene sequencing technology. The high-throughput technology is helpful to identify unexpected infectious causes of subacute or chronic CNS infection, including *Aspergillus* [9]. Therefore, patients with clinically suspected CNS infection should be tested by the wide coverage gene sequencing technology in the CSF to identify possible pathogens as far as possible.

Due to our inexperience, fluconazole was advised wrongly as a general treatment for fungal infections in the beginning, but it had no effect. After blood tests for *Aspergillus*, we confirmed that the patient had CNS aspergillosis. The American Society of Infectious Diseases recommended voriconazole as the first line treatment for aspergillosis [8]. Considering the mild clinical symptoms and immunocompetence of the patient, we gave him voriconazole alone for 12 weeks and achieved satisfactory outcomes.

In all, we revealed that aspergillosis infection of CNS can last for more than 20 years and the major symptom is only an intermittent throbbing headache in an immunocompetent patient. Vascular invasion is probably the mechanism of headache in our patients with CNS aspergillosis. We should enlarge the use of high-throughput gene sequencing technology in patients with suspected CNS infection to identify the pathogens as much as possible.

## Data Availability

All data generated and analyzed during this study are included in this article.

## Abbreviations

CNS: Central Nervous System
CSF: Cerebrospinal Fluid
GM: Galactomannan
ICP: Intracranial Pressure
NTS: Nanopore Targeted Sequencing
WBCs: White Blood Cells
